# Development and Validation of an Individualized Immune-Related Gene Pairs Prognostic Signature in Papillary Renal Cell Carcinoma

**DOI:** 10.3389/fgene.2020.569884

**Published:** 2020-11-09

**Authors:** Xianghong Zhou, Shi Qiu, Di Jin, Kun Jin, Xiaonan Zheng, Lu Yang, Qiang Wei

**Affiliations:** Department of Urology, Institute of Urology, National Clinical Research Center for Geriatrics and Center of Biomedical Big Data, West China Hospital of Sichuan University, Chengdu, China

**Keywords:** papillary renal carcinoma, immune-related gene pairs, prognostic signature, The Cancer Genome Atlas, gene expression omnibus

## Abstract

Papillary renal carcinoma (PRCC) is one of the important subtypes of kidney cancer, with a high degree of heterogeneity. At present, there is still a lack of robust and accurate biomarkers for the diagnosis, prognosis and treatment selection of PRCC. Considering the important role of tumor immunity in PRCC, we aim to construct a signature based on immune-related gene pairs (IRGPs) to estimate the prognostic of patients with PRCC. We obtained gene expression profiling and clinical information of patients with PRCC from The Cancer Genome Atlas (TCGA) and Gene Expression Omnibus (GEO), which were divided into discovery (*n* = 287) and validation (*n* = 28) cohorts, respectively. By univariate analysis, multivariate Cox analysis, and least absolute shrinkage and selection operator (Lasso) analysis, we selected 14 IRGPs with a panel of 22 unique genes to construct the prognostic signature. According to the signature, we stratified patients into high-risk group and low-risk group. In both discovery and validation cohorts, the results of Kaplan-Meier analysis showed that there were significant differences in OS between the two groups (*p* < 0.001). Combined with multiple clinical and pathological factors, the results of multivariate analyses confirmed that this signature was an independent predictor of OS (HR, 3.548; 95%CI, 2.096-6.006; *p* < 0.001). The results of immune infiltration analysis demonstrated that the abundance of multiple tumor-infiltration lymphocytes such as CD8 + T cells, Tregs, and T follicular cell helper were significantly higher in the high-risk group. Functional analysis showed that multiple immune-related signaling pathways were enriched in the high-risk group. In conclusion, we successfully established an individualized prognostic IRGPs signature, which can accurately assess and predict the OS of patients with PRCC.

## Introduction

Kidney cancer is one of the most common malignant tumors in the urinary system, and it is estimated that there will be 73,750 new cases diagnosed of kidney cancer in the United States in 2020 (Siegel et al., 2020). Papillary Renal Cell Carcinoma (PRCC) is a relatively rare histological subtype in kidney cancer, second to clear cell renal carcinoma (ccRCC), and accounts for about 10%–20% of kidney cancer (Courthod et al., 2015). PRCC is a heterogeneous disease, and the outcomes of different patients vary greatly in terms of disease progression, survival and response to therapy (Linehan et al., 2016). Currently, the common classification of PRCC is based on histology and mainly includes two main sub-types: PRCC type 1 and PRCC type 2 (Delahunt and Eble, 1997; Linehan et al., 2016). However, several previous studies have demonstrated that the classification has limited discrimination for the clinical outcomes of PRCC (Bigot et al., 2016; Crépel et al., 2009; Mejean et al., 2003). Meanwhile, due to the relatively small number of PRCC cases, many clinical and molecular studies on kidney cancer have not included patients with PRCC. Thus, in order to provide more specific and accurate biomarkers for the diagnosis, treatment and prognosis of PRCC, we need more researches on molecular profiling of PRCC to provide reliable data.

In recent years, immunotherapy based on immune checkpoint inhibitors (ICIs) has been applied in various tumors, greatly improving the survival outcomes of patients with advanced tumors. These ICIs include multiple agents that target programmed cell death protein 1 (PD-1), programmed death ligand 1 (PD-L1) or cytotoxic T lymphocyte associated protein 4 (CTLA4) (Del Paggio, 2018). For ccRCC, nivolumab, a PD-L1 inhabitor, has been approved for patients with metastatic tumors because of the encouraging results of random control trials (Motzer et al., 2015). It can be seen that the successful immunotherapy on ccRCC may also change the treatment of PRCC. Tumor immunology characteristics are closely related to the prognosis and treatment response of patients with PRCC. Prognostic markers based on immunology may help the risk stratification and treatment selection of patients with PRCC. Currently, there is few research teams to identify and validate a immune-related risk signature for prognosis of PRCC. More stable and reliable immune-related markers are still urgently needed.

To circumvent these defects, we utilized the data from The Cancer Genome Atlas (TCGA) as the discovery cohort to construct an immune-related gene pairs (IRGPs) prognostic signature for predicting overall survival (OS) of PRCC patients. The data from Gene Expression Omnibus (GEO) as the external validation cohort set was used to verify the reliability of the signature.

## Materials and Methods

### Data Collection and Processing

This was a retrospective study based on two independent datasets. The first dataset was the discovery cohort including 287 samples from TCGA. The second dataset was the validation cohort including 28 samples from GEO. The gene expression quantification data and clinical data of these samples were obtained from TCGA^1^, GEO^2^ and related publication (Yang et al., 2005). A total of 315 samples were included in our analyses. All the samples contained completed follow-up information.

For the data from TCGA, the transcriptome profiling was obtained by RNA-seq and measured by Fragments per Kilobase Million (FPKM) values and genes with zero of FPKM values in more than half of the samples were removed. As for the data from GEO, the transcriptome profiling was converted from the probe level to the corresponding gene symbol according to the annotation file. Any samples without complete follow-up information would be excluded.

### Construction of Immune-Related Gene Pairs Prognostic Signature

Firstly, we obtained information of 2,498 immune-related genes (IRGs) from the ImmPortdatabase^3^. These IRGs were related to natural killer cell cytotoxicity, cytokines, cytokine receptors, antigen processing, T-cell receptor signaling pathway, B-cell antigen receptor signaling pathway and so on. We measured the IRGs involved in this study, and only IRGs with a median absolute deviation (MAD) greater than 0.5, that is, with a large degree of variation, were retained. Then, for each IRG, we composed a series of unique immune-related gene pairs (IRGPs) with each of the other IRGs to make pairwise comparisons. One IRGP contained two IRGs. The first gene in the IRGP was named as IRG1, and the second gene in the IRGP was named IRG2. The value of each IRGP was determined by the relative ranking of the expression levels of the two IRGs that made up the IRGP. If the expression level of the IRG1 was higher than the latter IRG2, then the value of this IRGP was considered to be 1, otherwise it is 0. After removing IRGPs with small variation and imbalanced distribution, the remaining IRGPs were used as candidate signatures to predict the OS of PRCC.

Subsequently, log-rank test was used to preliminarily assess the correlation between IRGPs and the OS of PRCC patients in the discovery cohort. Then, we applied a Cox proportional hazards regression model combined with the least absolute shrinkage and selection operator (Lasso) and 10-fold cross validation to minimize the risk of overfitting. After the above two screenings, the remaining IRGPs were used as the prognostic IRGPs to predict the OS of PRCC patients. Finally, using the values and coefficients of these prognostic IRGPs, we could build a model for calculating the immune-related Gene Pairs index (IRGPI) risk score of each sample. The IRGPI risk score signature was calculated as follows:

IRGPI=(Value×g⁢e⁢n⁢ep⁢a⁢i⁢r-1Coef)g⁢e⁢n⁢ep⁢a⁢i⁢r-1+(Value×g⁢e⁢n⁢ep⁢a⁢i⁢r-2Coef)g⁢e⁢n⁢ep⁢a⁢i⁢r-2+…+(Value×g⁢e⁢n⁢ep⁢a⁢i⁢r-nCoef)g⁢e⁢n⁢ep⁢a⁢i⁢r-n.

As described above, *Value* is the value of the gene pair which is determined by the relative expression level of IRG1 and IRG2. And *Coef* is the gene pair’s regression coefficient derived from the LASSO regression model. According to the score calculated by the risk signature model, the samples were divided into high-risk and low-risk groups. The optimal cut-off of the scores was determined using time-dependent ROC at 1 year in the TCGA dataset for overall survival (OS) (Zuo et al., 2018).

To separate patients into low or high-risk groups, time-dependent receiver operating characteristic (ROC) curve was used to find the optimal cut-off value of IRGPI at 1 year in the training cohort for OS. The point closest to the 100% true positive rate and 0% false-positive rate could be seen as the cut-off point.

### Validation of the IRGPs Prognostic Signature

In order to verify the accuracy of IRGPs signature on the stratification of patients’ prognosis, we calculated the IRGPI risk score of each sample of the discovery cohort (TCGA) and the validation cohort (GSE2748) separately, and divided them into different risk groups according to the cut-off value. Kaplan-Meier curve and log-rank test were used to verify whether the OS between the two groups was significantly different. In both discovery cohort and validation cohort, we calculated the area under the curve (AUC) to evaluate the prognostic accuracy of the immune−related risk signature, which ranges from 0 to 1 and 0.5 represents a random prediction (Heagerty et al., 2000).

Further, for confirming that the IRGPs signature was an independent prognostic factor, we combined the IRGPs signature with available clinicopathological in univariable and multivariable Cox proportional hazards regression model analyses, including age, gender, stage and PRCC type.

Meanwhile, based on the samples from TCGA database, we furtherly tested the diagnostic performance of the IRGPs signature on distinguishing ccRCC vs. PRCC, type 1 PRCC vs. type 2 PRCC and healthy sample vs. PRCC sample. The method of evaluating the diagnostic performance was to plot the ROC curve and calculate the AUC.

In addition, we also performed the differential gene expression analysis between the high-risk and low-risk groups based on our signature. This analysis was performed with the R package LIMMA (linear models for microarray data). Each gene expression was calculated based on the false discovery rate (FDR) < 0.05.

### Analysis of Correlation Between IRGPs Signature and Immune Cells Infiltration

Tumor-infiltrating lymphocytes (TILs) in PRCC samples were assessed by applying the “Cell type Identification By Estimating Relative Subsets Of RNA Transcripts (CIBERSORT)” deconvolution algorithm (Newman et al., 2015). By analyzing the relative expression levels of 547 genes in samples, CIBERSORT could predict the proportion of 22 types of TILs in each PRCC sample. The gene expression signature matrix of 22 tumor-infiltrating immune cells was obtained from the CIBERSORT platform^4^. We set 1000 permutations and *P* < 0.05 as the criteria for the successful deconvolution of a sample. Then, we compared the proportions of the immune cell subsets between the IRGPI risk groups using the Mann–Whitney U test.

### Gene Ontology and Gene Set Enrichment Analysis

GO analysis was performed for enhancing the biological understanding of the prognostic IRGPs signature. GSEA was conducted using the Bioconductor package “fgsea” with 100,000 permutations. We obtained and compared the log2 fold change between the gene expression profiles of different IRGPI risk groups. All the biological processes involved in our study were obtained from the Molecular Signature Database (MSigDB C5 databases, version 7). Gene sets with FDR-adjusted *P* < 0.05 or nominal (NOM) *P* < 0.05 were selected.

## Statistical Analysis

The statistical software R (version 3.6.2), Perl (version 5.24.3) were used in the above analyses. Wilcox tests were used to compare the differences between two groups. A *p*-value < 0.05 was considered statistically significant.

## Results

### IRGPs Prognostic Signature Construction

A total of 315 patients with PRCC were included in our study. We assigned the samples from TCGA (*n* = 287) to the discovery cohort and the samples from GEO (*n* = 28) to the validation cohort. Then we obtained a list of 2,498 immune-related genes (IRGs) from the ImmPortdatabase. Genes with relatively lower degree of variation (MAD ≤ 0.5) were removed firstly. After preliminary filtering in the discovery cohort and validation cohort, 172 IRGs were retained. Based on the 172 IRGs, we successfully constructed 3683 IRGPs as candidates. We evaluated the correlation between all IRGPs and OS using univariate Cox analysis, of which there were 48 significant prognostic IRGPs. Furtherly, we used Lasso Cox proportional hazard regression on the discovery cohort, and finally selected 14 IRGPs with more stable prognostic significance to construct the model for calculating the IRGPI (Table 1). The actual formula for calculating the IRGPI risk scores was as follows: IRGPI = (Value_*CTSS| ADM*_ ×−0.13784168) + (Value_*HLA–DPA*__1__| IFITM__1_ ×−0.536023127) + (Value_*HSPA*__2__| *NR*__2__*F*__1_ ×−0.813388891) + (Value_*MICB*__| *CX*__3__*CR*__1_ × 0.547230703) + (Value_*RBP*__4__| *TNFRSF*__19_ ×−0.479900288) + (Value_*NOX*__4__| TNFSF__13__*B*_ ×−0.536067102) + (Value_*CHIT*__1__| CCL__4_ ×−0.055801169) + (Value_*VEGFA| AR*_ × 0.295949021) + (Value_*VEGFA| ITGB*__2_ × 0.313650746) + (Value_*ITGAV| TNFSF*__13_ × 0.033017443) + (Value_*WNT*__5__*A| NR*__2__*F*__1_ ×−0.421788958) + (Value_*BTK| TNFSF*__13__*B*_ ×−0.212282282) + (Value_*IFITM*__1__| TNFSF__13_ × 0.012878529) + (Value_*TNFSF*__13__*B| CSF*__3__*R*_ × 0.448335019).

**TABLE 1:** Detail information about 14 immune-related gene pairs used to calculate immune-related gene pairs index.

IRG 1	Immune process	IRG 2	Immune process	Coefficient
CTSS	Antigen_Processing_and_ Presentation	ADM	Antigen_Processing_and_Presentation	−0.13784168
HLA-DPA1	Antigen_Processing_and_ Presentation	IFITM1	BCRSignalingPathway	−0.536023127
HSPA2	Antigen_Processing_and_ Presentation	NR2F1	Cytokine_Receptors	−0.813388891
MICB	Antigen_Processing_and_ Presentation	CX3CR1	Chemokine_Receptors	+0.547230703
RBP4	Antimicrobials	TNFRSF19	Cytokine_Receptors	−0.479900288
NOX4	Antimicrobials	TNFSF13B	Cytokines	−0.536067102
CHIT1	Antimicrobials	CCL4	Antimicrobials	−0.055801169
VEGFA	Antimicrobials	AR	Cytokine_Receptors	+0.295949021
VEGFA	Antimicrobials	ITGB2	NaturalKiller_Cell_ Cytotoxicity	+0.313650746
ITGAV	Antimicrobials	TNFSF13	Cytokines	+0.033017443
WNT5A	Antimicrobials	NR2F1	Cytokine_Receptors	−0.421788958
BTK	Antimicrobials	TNFSF13B	Cytokines	−0.212282282
IFITM1	BCRSignalingPathway	TNFSF13	Cytokines	+0.012878529
TNFSF13B	Cytokines	CSF3R	Cytokine_Receptors	+0.448335019

*IRG: immune-related gene.*

The 14 IRGPs included a panel of 22 unique genes, and main of them were associated with antigen processing and presentation, antimicrobials, and cytokines. Based on the Metascape database^5^, we found a lot of immune-related signaling pathways significantly enriched within the 22 unique genes. For example, Cytokine-cytokine receptor interaction and positive regulation of immune effector process were the two most enriched pathways, and they were also two pathways closely related to immunity (Supplementary Figure S1).

**FIGURE 1:**
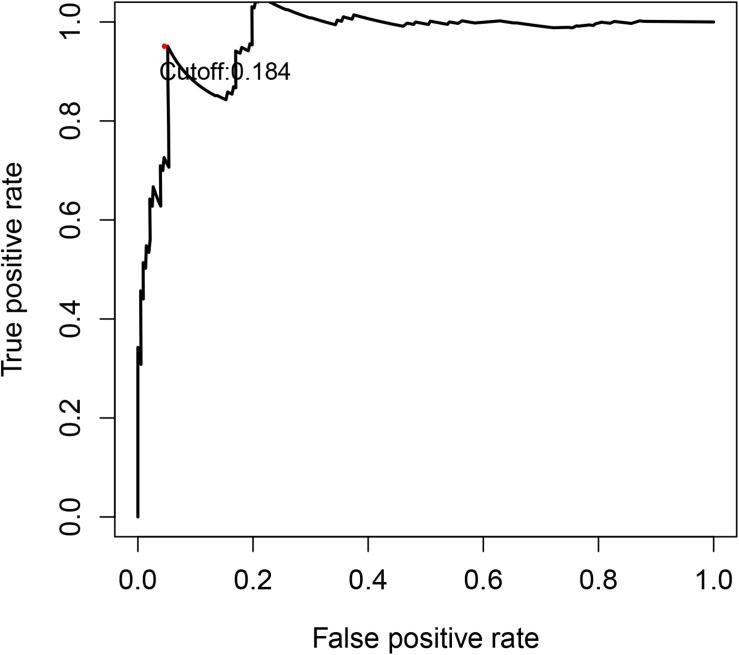
Time-dependent ROC curve for IRGPI risk score in the discovery cohort. 0.184 was used as a cut-off for IRGPI risk score to stratify patients into low- or high-risk groups. ROC, receiver operating curve; IRGPI, immune-related gene pair index.

After calculating the IRGPI risk score of each patient, according to the time-dependent ROC curve analysis, the cut-off value for distinguishing patients into high- or low-risk groups was determined to be 0.184 (Figure 1 and Supplementary Table S1). For example, we can detect the expression values of the 22 unique gene in a certain patient. Based on the relative ranking of the gene expression level, we can obtain the 14 IRGPs. According to the IRGPI risk score formula as described above, the risk score was calculated. Compared with the cut-off value of risk score of 0.184, we can assign the patient into low-risk or high-risk groups can be determined. After calculating the risk scores for all patients in the cohorts, we successfully divided the patients into high-risk groups and low-risk groups based on the cut-off value of 0.184, and in the following analyses, we verified whether the high- and low-risk groups based on our signature was consistent with the patient’s clinical OS outcome.

### Validation of the IRGPs Signature as an Independent Prognostic Factor

**FIGURE 2:**
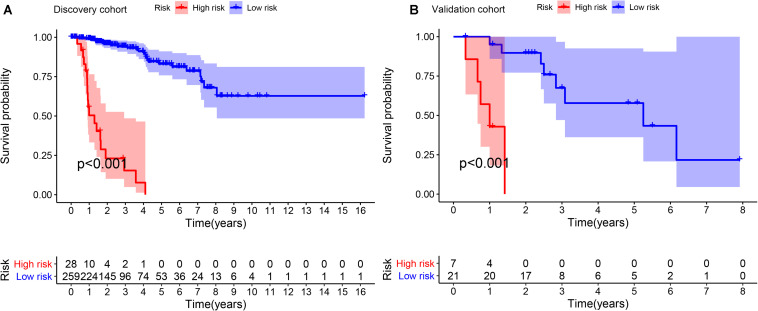
Kaplan-Meier survival curves of OS between different high- and low- risk groups in discovery cohort **(A)** and validation cohort **(B)**. OS: overall survival; IRGPI: immune-related gene pair index.

**FIGURE 3:**
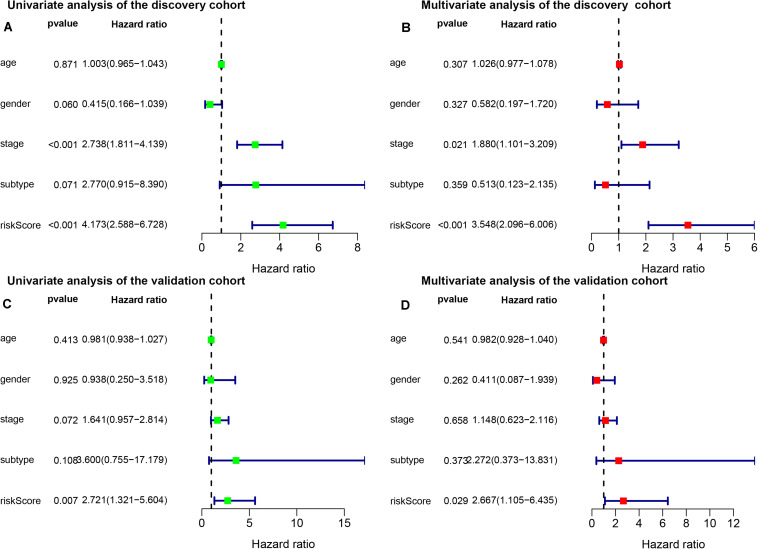
Univariate **(A)** and multivariate **(B)** analyses of prognostic factors in discovery cohort and univariate **(C)** and multivariate **(D)** analyses of prognostic factors in validation cohort identified the signature was an independent prognostic factor. Subtype, histological subtypes of papillary renal carcinoma; riskScore, immune-related gene pair index (IRGPI) risk score.

In the discovery cohort, Kaplan-Meier curves showed that the OS of the patients in high-risk group was significantly poorer than that of the patients in low-risk group (*p* < 0.001) (Figure 2A). Subsequently, we jointly evaluated the effects of IRGPI risk score, age, gender, stage, and PRCC type on OS in univariate and multivariate Cox regression analyses. The results of univariate analysis showed that the risk score based on IRGPs signature was significantly associated with the patient’s prognosis (HR, 4.173; 95%CI, 2.588-6.728; *p* < 0.001). And the results of the multivariate analyses furtherly demonstrated that IRGPs signature was an independent prognostic factor independent of age, gender, stage, and PRCC type (HR, 3.548; 95%CI, 2.096-6.006; *p* < 0.001) (Figures 3A,B). The results of AUC demonstrated the excellent predictive accuracy of the signature for OS of PRCC patients (1-year AUC, 0.957; 3-year AUC, 0.825; 5-year AUC, 0.760).

To validate the consistency of prognostic value of the IRGPs signature, we applied it in an independent validation cohort from GSE2748 (*n* = 28). Similarly, we calculated the IRGPI risk score for each patient in the validation cohort and stratified patients according to the cut-off value obtained in the discovery cohort (Supplementary Table S1). Same as the previous result, the high-risk group was associated with poorer OS than the low-risk group (*p* < 0.001) (Figure 2B). The results of univariate and multivariate Cox regression analyses demonstrated IRGPI risk score was the independent prognostic factor again (Univariate: HR, 2.721; 95%CI, 1.321-5.604; *p* = 0.007; and Multivariate: HR, 2.667; 95%CI, 1.105-6.435; *p* = 0.029) (Figures 3C,D). The accuracy of the application of the IRGPs signature in the validation cohort was still promising (1-year AUC, 0.786; 3-year AUC, 0.791; 5-year AUC, 0.820).

From the TCGA database, we obtained 539 ccRCC samples and 32 healthy samples to test the additional diagnostic performance of our signature (Supplementary Figure S2). The results showed that our signature performed better in distinguishing ccRCC and PRCC (AUC = 0.876), as well as healthy samples and PRCC samples (AUC = 0.823). In terms of distinguishing between type 1 PRCC and type 2 PRCC, our signature performance was average (AUC = 0.685). This demonstrated that our signature had potential clinical value in kidney cancer classification and risk stratification.

In total, 1281 differentially expressed genes were obtained through our analyses, which include 681 upregulated genes and 600 downregulated genes. In order of the magnitude FDR, Supplementary Table S3 showed the top ten differentially expressed genes that were up- or down-regulated. The overall differential expression was shown in Supplementary Figure S3.

### Immune Cells Infiltration in Different Risk Groups

**FIGURE 4:**
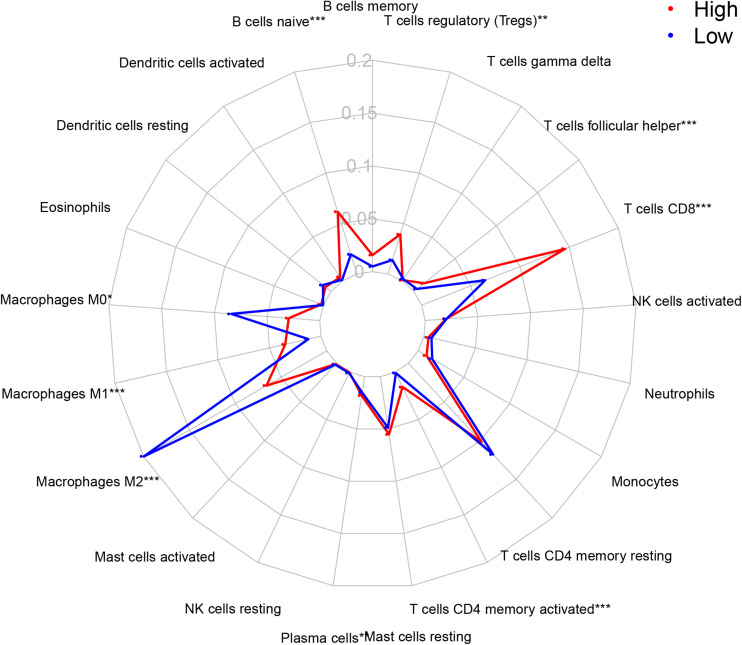
The proportion of 22 TILs in tumor immune microenvironment in high- and low- risk groups. TILs, tumor-infiltration lymphocytes; IRGPI, immune-related index; *p < 0.05; **p < 0.01; **p < 0.001; T cells CD4 + naive is not shown in the picture because of its low abundant.

Based on CIBERSOFT algorithm, we systematically estimated the proportions of 22 kinds of TILs for each PRCC patient in different risk groups. Detailed information of the output of the algorithm was shown in Figure 4. We found that different TILs were significantly enriched in different risk groups. In the high-risk group, T CD8^+^ cells (*p* < 0.001), T regulatory cells (Tregs, *p* = 0.001), T follicular helper cells (*p* < 0.001), B cells naive (*p* < 0.001), Plasma cells (*p* = 0.009), T CD4^+^ memory cells activated (*p* < 0.001), Macrophage M1 (*p* < 0.001) were highly expressed, while Macrophage M0 (*p* = 0.027), Macrophages M2 (*p* < 0.001) were lowly expressed, compared with the low-risk group.

### Functional Assessment of the IRGPs Signature

**FIGURE 5:**
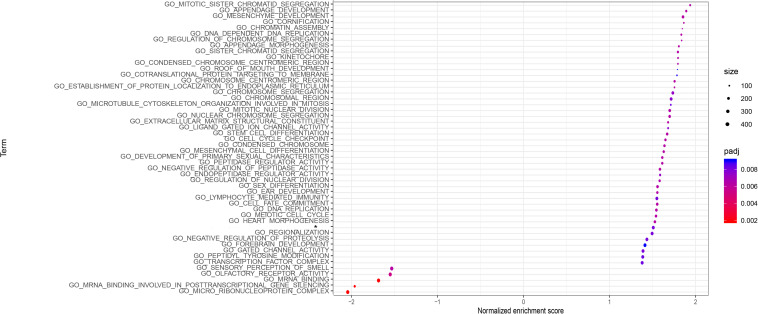
GO analysis of the 22 immune signature genes. The top 50 GO terms with FDR < 0.05 are shown in the figure. GO, gene oncology; FDR, false discovery rate.

**FIGURE 6:**
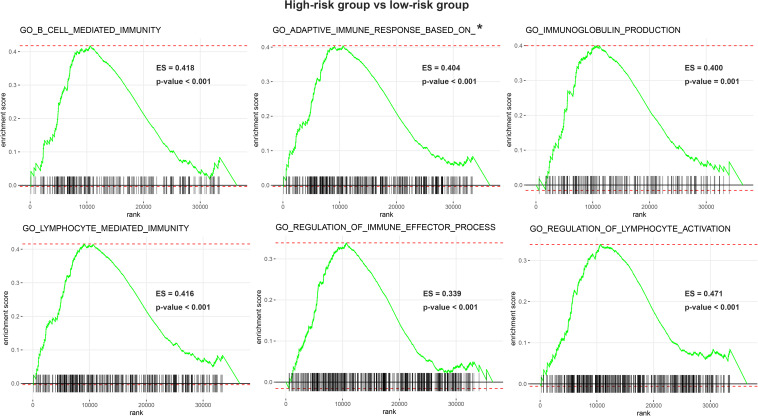
Enrichment plots of gene set enrichment analysis (GSEA). GESA found 6 immune-related pathways with significant differences between two groups. GSEA, gene set enrichment analysis; IRGPI, immune-related index; *GO_ADAPTIVE_IMMUNE_RESPONSE_BASED_ON_SOMATIC_RECOMBINATION_OF_ IMMUNE_RECEPTORS_BUILT_FROM_IMMUNOGLOBULIN_SUPERFAMILY_DOMAINS.

We performed GO analysis and GSEA for functional annotation of the IRGPs signature (Supplementary Table S2). Figure 5 showed a total of top 50 GO terms with FDR < 0.05, sorted by FDR. We found that the IRGPs signature genes in the discovery cohort were mostly involved in “mitotic sister chromatid segregation.” The results of GSEA demonstrated multiple immune-related pathways that differed between high- and low-risk groups significantly, including “adaptive immune response based on somatic recombination of immune receptors built from immunoglobulin superfamily domains,” “lymphocyte mediated immunity,” “B cell mediated immunity,” “immunoglobulin production,” “regulation of immune effector process,” and “regulation of lymphocyte activation” (Figure 6). Thus, the IRGPs signature demonstrated an intensive immune phenotype.

## Discussion

Considering the important impact of tumor immunity in PRCC, our study analyzed a discovery cohort from TCGA to establish a robust prognostic signature based on 14 immune-related gene pairs for predicting OS of PRCC patients. The signature can accurately distinguish the prognosis of patients with PRCC and is a prognostic factor independent of other clinical pathological factors. An external validation cohort from GEO confirmed the reliability of the prognostic signature. Furtherly, we found that the signature was associated with various proportion of specific TILs in the tumor immune microenvironment, and is involved in many immune-related GO terms. The prognostic signature can be used as an important marker for risk stratification in patients with PRCC, and may be a potential target for immunotherapy.

In view of the obvious heterogeneity of PRCC, some research groups have studied the methods of classification or prognostic stratification of PRCC. At present, the most widely used PRCC classification method divides PRCC into type 1 and type 2 according to histological characteristics. Type 1 is characterized by papillary and tubular structures covered by small cells containing a basophilic cytoplasm and a small uniform oval nucleus, while type 2 is characterized by Large cells with eosinophilic cytoplasm and large spherical nuclei (Delahunt and Eble, 1997; Zbar et al., 1994). However, the role of histological sub-types of PRCC in distinguishing patients from different clinical outcomes is controversial. A multicenter retrospective study included 486 patients undergoing partial nephrectomy with the two PRCC histological sub-types (76% type 1 and 24% type 2). The results showed that there were no demographic, clinical or tumor differences between the two types of PRCC (Bigot et al., 2016). Another research group performed a retrospective study of 88 PRCC patients and studied the prognostic factors of PRCC. The results of multivariate analysis demonstrated that the stage and grade were independent prognostic factors, excluding histological sub-types (Mejean et al., 2003). Our findings confirmed the results of previous studies again.

In both the discovery cohort and the validation cohort, the results of univariate and multivariate analyses suggested that the histological sub-types of PRCC were not independent prognostic factors for OS of patients with PRCC, while our IRGPs signature shows a robust independent prognostic ability. Therefore, although we already knew, different gene mutations had been associated with the 2 papillary histological sub-types, including FH gene with type 2 and c-met with type 1, the molecular factors that determine the clinical manifestations of tumors still required more exploration (Schmidt et al., 1997; Tomlinson et al., 2002).

Some researchers have developed several signatures on the prognosis of PRCC at the molecular level, including mRNA, lncRNA, alternative splicing, mutation and etc., (Cao et al., 2020; Duan and Zhang, 2019; Zhang C. et al., 2019; Zuo et al., 2018). Previous study reported a five-gene signature to predict overall survival of patients with PRCC (Gao et al., 2019). The researchers identified the differentially expressed mRNAs between cancer and normal tissues and constructed the signature based on these mRNA. The signature could distinguish patients with different survival outcomes, and the results were statistically significant. Another study reported a methylation-driven genes related signature (Liu et al., 2020). The authors tried to screen the biomarkers of pRCC from methylation-driven genes through bioinformatics methods. They finally constructed a signature based 7 methylation-driven genes which were significantly associated with patients’ survival. However, these two signatures have not yet reached a robust high accuracy rate, and have not considered the important impact of tumor immunology on the prognosis and treatment of PRCC. Some researchers have begun to pay attention to the role of tumor immunology on the prognosis of PRCC. A signature based on immune-related genes of PRCC has recently been reported (Wang et al., 2019). The authors constructed a prognostic signature of 15 immune genes to predict the survival outcome of PRCC patients, showing the value of immune-related prognostic signatures in PRCC. Summarize the above three recently published PRCC signatures, there were still some deficiencies. The three signatures all used the data from TCGA, and they lacked external validation cohort. At the same time, these studies also lack multivariate analysis including histological sub-types. In our research, we further added important independent external validation sets to make the signature more robust, and at the same time proved the signature’s prognostic role independent of age, gender, stage, and PRCC histological sub-types. As far as the accuracy of prediction was concerned, compared with the 15 immune genes signature, our signature had an advantage in predicting AUC of 1, 3, and 5 years (0.957, 0.825, 0.760 vs. 0.934, 0.796, 0.662). In addition, our signature was built on gene pairs, and this method had some additional advantages. The biological heterogeneity of tumors and differences in sequencing platforms often caused technical bias, thus standardizing gene expression profiles was necessary and difficult. We used a novel method based on gene pairs to construct the prediction model. Data preprocessing such as scaling and normalization was not required, instead, we compared the relative ranking and pairing of gene expression values. This method could reduce the impact of the technical bias of different platforms on the results and improve the robustness of the signature (Eddy et al., 2010). Meanwhile, data from different platforms, data from different periods of the same platform, data from different reagents in the same sample, and data from the same sample at different times, etc., will often produce batch effects. Batch effects may have an impact on the results of the research. Similarly, the IRGP signature based on the relative expression levels of genes in each sample can be used to reduce batch efforts (Sun et al., 2020). In recent years, this method has been applied to the construction of various tumor prognosis models with excellent results, including non-small cell lung cancer, colorectal cancer, and serous ovarian carcinoma (Li et al., 2017; Wu et al., 2019; Zhang L. et al., 2019).

The prognostic signature was construct of 14 IRGPs with 22 unique genes. Most of this signature gene were related to antigen processing and presentation, and cytokines, and were enriched in multiple immune-related GO terms. Among the 22 unique genes, previous studies demonstrated NRF2A was an important gene regulating tumor cell dormancy. Down-regulated NRF2A was associated with the occurrence and recurrence of various tumors (Borgen et al., 2018; Sosa et al., 2015). In ccRCC, high expression of APRIL (TNFSF13) was closely related to poor prognosis, and VEGFA was significantly upregulated compared with normal tissue (Lee et al., 2015; Wang et al., 2018). High expression of CTSS is a predictor of poor prognosis and tumor metastasis in papillary carcinoma of the thyroid (Tan et al., 2018). Meanwhile, overexpression of HSPA2 was related to tumor angiogenesis and poor prognosis of pancreatic cancer, while the survival prognosis of breast cancer patients with high expression of NOX4 was poor, too (Zhai et al., 2017; Ham et al., 2018). By the CIBERSORT algorithm, we found that some TILs’ proportions were significantly different between the two risk groups.

Between the two risk groups, we observed significant differences in the proportion of specific TILs. There were some interesting findings. In general, CD8^+^ T cells can recognize tumor specific antigens and played an important role in tumor immunity. Higher CD8^+^ T cell infiltration in multiple cancer types is associated with a better prognosis (Chen and Mellman, 2013). However, in our study, we could find that the high-risk group had obvious higher CD8^+^ T cell infiltration than the low-risk group did. There are some research results that can explain this rare phenomenon to some degrees. First, previous research found that CD8^+^ T cells were not only specific for tumor-derived antigens, but also specific for non-tumor antigens. The enrichment of CD8^+^ T cells may not always play an anti-tumor effect, and has phenotypic heterogeneity in tumors and patients. Therefore, the prognostic effect of CD8 + T cells is not necessarily the same in different tumor types and patients (Simoni et al., 2018). Meanwhile, At the same time, some researchers also found similar results in this study of RCC, that was, higher CD8^+^ T cell infiltration was associated with poor prognosis. The possible reason is the dysfunction of CD8 + T cells caused by various factors, such as high DGK-alpha, disabled MAPK pathways and JAK3/STAT5/6 pathway alterations (Prinz et al., 2012). And studies have confirmed that abnormal dendritic cells are involved in the process of CD8^+^ T cell suppression, which may cause CD8^+^ T cell to have higher abundance, but not to exert the corresponding anti-tumor function (Giraldo et al., 2015; Pan et al., 2020). In addition, researchers have found that the abundance of M2 macrophages and the abundance of CD8 + T cells in RCC are negatively correlated, which supports our findings (Pan et al., 2020). In our study, the M2 macrophage abundance of the high-risk group was significantly lower than that of the low-risk group. Similarly, the abundance of Treg cell and T cells follicular helper in the high-risk group was significantly higher than those in the low-risk group. These two TILs are considered to be factors that promote tumor progression and are related to the poor prognosis of patients (Finotello and Trajanoski, 2017; Long et al., 2019). Previous publication found that the abundance of CD8^+^ T cells was positively correlated with the abundance of Tregs and T cells follicular helper, and negatively correlated with the abundance of M2 macrophages, which is consistent with our research results (Pan et al., 2020). Macrophage M2, T regs and T cell follicular helper may play a role in the balance of the exhaustion or inhibition of T cells, and balance each other (Speiser et al., 2014).

There are still some limitations to our study. First, although we have tried to introduce an external validation to improve the robustness of our results, our research is still retrospective in nature. In the future, we need more prospective research to further apply and verify our findings. Second, our research data is based on RNA-seq and microarray, the high price and complicated analysis process limit the clinical application of our results. We need more researches to explore how to simplify the IRGPs signature and how to combine it with existing clinical pathological factor to improve the ease of use and accuracy of clinical applications.

## Conclusion

All in all, we have established an individualized prognostic immune-related gene pairs signature, which can accurately assess and predict the OS of patients with PRCC. The signature we developed is an independent prognostic factor, a practical tool for stratifying the prognosis risk of patients, and may provide a reference when screening PRCC patients to receive immunotherapy.

## Data Availability Statement

The datasets generated and analysed during the current study are available in the TCGA-KIRP, (https://portal.gdc.cancer.gov/) and GSE2748, (http://www.ncbi.nlm.nih.gov/geo/).

## Ethics Statement

Ethical review and approval was not required for the study on human participants in accordance with the local legislation and institutional requirements. Written informed consent for participation was not required for this study in accordance with the national legislation and the institutional requirements.

## Author Contributions

QW and LY designed the study. XhZ and SQ were responsible for writing, collecting data, analysis, interpretation, and revision present article. DJ and KJ were responsible for data collecting and analysis partly. XnZ was responsible for data analysis partly. All authors have read and approved the final manuscript.

## Conflict of Interest

The authors declare that the research was conducted in the absence of any commercial or financial relationships that could be construed as a potential conflict of interest.

## Footnotes

1 https://portal.gdc.cancer.gov/

2 https://www.ncbi.nlm.nih.gov/geo/query/acc.cgi?acc=GSE2748

3 https://immport.niaid.nih.gov

4 https://cibersortx.stanford.edu/

5 www.metascape.org

## Supplementary Material

Supplementary Figure 1 Signaling pathways significantly enriched withing the 22 unique identified genes contained in the immune-related genes pairs.

Supplementary Figure 2 Time-dependent receiver operating characteristic (ROC) curve for testing the diagnostic performance of the IRGPs signature on healthy samples vs. PRCC (a), ccRCC vs. PRCC (b), and type 1 PRCC vs. type 2 PRCC. PRCC, papillary renal cell carcinoma; ccRCC, clear cell renal cell carcinoma.

Supplementary Figure 3 Gene expression differences in high- and low-risk groups based on IRGP signature. The x-axis represents the difference between the two data sets following log2 conversion and the y-axis represents the *p*-value (log 10).

## Abbreviations

AUC: the area under the curve
ccRCC: clear cell renal carcinoma
CI: confidence interval
GEO: Gene Expression Omnibus
HR: hazard ratio
IRG: immune-related Gene
IRGPI: immune-related Gene Pairs index
IRGPs: immune-related gene pairs
Lasso: least absolute shrinkage and selection operator
MAD: median absolute deviation
OS: overall survival
PRCC: Papillary renal carcinoma
ROC: receiver operating characteristic
TCGA: The Cancer Genome Atlas
TILs: tumor-infiltration lymphocytes.
