# Supplementary material for: Development and Validation of an Individualized Immune-Related Gene Pairs Prognostic Signature in Papillary Renal Cell Carcinoma
Source: Front Genet. 2020 Nov 9;11:569884. doi: 10.3389/fgene.2020.569884 (PMC7680997; doi:10.3389/fgene.2020.569884)
Supplement: Supplementary file 1 [file Data_Sheet_1.docx]

**Table S1 Detail information of the immune risk stratification**

| **ID** | **Cohort** | | **Risk score** | **Risk group** | |
| --- | --- | --- | --- | --- | --- |
| TCGA-J7-A8I2 | TCGA | 0.722826 | | | high |
| TCGA-2Z-A9J1 | TCGA | -3.19309 | | | low |
| TCGA-B9-4116 | TCGA | -0.4648 | | | low |
| TCGA-UZ-A9PS | TCGA | -1.61607 | | | low |
| TCGA-O9-A75Z | TCGA | -2.04112 | | | low |
| TCGA-Y8-A8S1 | TCGA | -1.85945 | | | low |
| TCGA-BQ-7060 | TCGA | -3.1472 | | | low |
| TCGA-IA-A83S | TCGA | -2.3649 | | | low |
| TCGA-MH-A560 | TCGA | -2.04034 | | | low |
| TCGA-5P-A9K9 | TCGA | 0.695433 | | | high |
| TCGA-Y8-A8RY | TCGA | -3.19309 | | | low |
| TCGA-A4-7996 | TCGA | -3.13729 | | | low |
| TCGA-AL-7173 | TCGA | -1.53858 | | | low |
| TCGA-B9-7268 | TCGA | -2.53248 | | | low |
| TCGA-EV-5903 | TCGA | -1.47802 | | | low |
| TCGA-2Z-A9JJ | TCGA | -0.16105 | | | low |
| TCGA-MH-A855 | TCGA | -2.16429 | | | low |
| TCGA-B9-A5W7 | TCGA | -2.50802 | | | low |
| TCGA-IZ-A6M9 | TCGA | -2.64586 | | | low |
| TCGA-2Z-A9JP | TCGA | -2.22407 | | | low |
| TCGA-SX-A7SR | TCGA | -3.19309 | | | low |
| TCGA-HE-A5NH | TCGA | -3.19309 | | | low |
| TCGA-BQ-7055 | TCGA | 0.163764 | | | low |
| TCGA-BQ-7058 | TCGA | 1.114994 | | | high |
| TCGA-BQ-5891 | TCGA | -1.99677 | | | low |
| TCGA-GL-8500 | TCGA | -3.13729 | | | low |
| TCGA-F9-A7VF | TCGA | -1.70391 | | | low |
| TCGA-BQ-7053 | TCGA | -1.44787 | | | low |
| TCGA-SX-A71R | TCGA | -1.38685 | | | low |
| TCGA-B1-A654 | TCGA | -2.31102 | | | low |
| TCGA-2Z-A9J7 | TCGA | -0.85115 | | | low |
| TCGA-BQ-5889 | TCGA | 0.719669 | | | high |
| TCGA-AL-3467 | TCGA | -0.66741 | | | low |
| TCGA-DW-7837 | TCGA | -3.19309 | | | low |
| TCGA-DW-7842 | TCGA | -2.99945 | | | low |
| TCGA-2Z-A9JL | TCGA | -1.29474 | | | low |
| TCGA-5P-A9KA | TCGA | -1.79779 | | | low |
| TCGA-UZ-A9PM | TCGA | -2.41724 | | | low |
| TCGA-UZ-A9PX | TCGA | -2.3797 | | | low |
| TCGA-B1-A47O | TCGA | -1.34952 | | | low |
| TCGA-J7-8537 | TCGA | 1.199569 | | | high |
| TCGA-MH-A856 | TCGA | -0.81382 | | | low |
| TCGA-P4-AAVO | TCGA | -1.56126 | | | low |
| TCGA-B9-4113 | TCGA | -3.19309 | | | low |
| TCGA-B9-A8YI | TCGA | -1.26573 | | | low |
| TCGA-BQ-5890 | TCGA | -1.0953 | | | low |
| TCGA-UN-AAZ9 | TCGA | -0.90023 | | | low |
| TCGA-A4-A57E | TCGA | 0.355481 | | | high |
| TCGA-IA-A83T | TCGA | -3.19309 | | | low |
| TCGA-B9-4115 | TCGA | -3.19309 | | | low |
| TCGA-A4-A5DU | TCGA | -2.98081 | | | low |
| TCGA-4A-A93Y | TCGA | -0.57587 | | | low |
| TCGA-EV-5902 | TCGA | -2.99945 | | | low |
| TCGA-MH-A55W | TCGA | -2.98081 | | | low |
| TCGA-Y8-A8S0 | TCGA | -3.19309 | | | low |
| TCGA-MH-A561 | TCGA | -3.18021 | | | low |
| TCGA-BQ-7061 | TCGA | -0.92798 | | | low |
| TCGA-4A-A93W | TCGA | -2.3797 | | | low |
| TCGA-BQ-7049 | TCGA | -0.15522 | | | low |
| TCGA-SX-A7SO | TCGA | -2.38985 | | | low |
| TCGA-IA-A40Y | TCGA | -0.70632 | | | low |
| TCGA-P4-A5E7 | TCGA | -2.02795 | | | low |
| TCGA-GL-6846 | TCGA | -1.99677 | | | low |
| TCGA-BQ-7056 | TCGA | -2.10849 | | | low |
| TCGA-A4-A6HP | TCGA | -2.59006 | | | low |
| TCGA-G7-A8LC | TCGA | -2.45222 | | | low |
| TCGA-B1-5398 | TCGA | -3.0914 | | | low |
| TCGA-KV-A74V | TCGA | -1.35257 | | | low |
| TCGA-HE-A5NK | TCGA | -2.1029 | | | low |
| TCGA-P4-A5EB | TCGA | -3.19309 | | | low |
| TCGA-SX-A71V | TCGA | -3.19309 | | | low |
| TCGA-BQ-5876 | TCGA | 0.902756 | | | high |
| TCGA-B9-A69E | TCGA | -1.58011 | | | low |
| TCGA-UZ-A9Q1 | TCGA | -2.15424 | | | low |
| TCGA-5P-A9JZ | TCGA | -3.19309 | | | low |
| TCGA-5P-A9K0 | TCGA | -3.19309 | | | low |
| TCGA-SX-A7SQ | TCGA | -3.13729 | | | low |
| TCGA-2Z-A9JI | TCGA | -2.31518 | | | low |
| TCGA-MH-A854 | TCGA | -1.972 | | | low |
| TCGA-A4-7288 | TCGA | -2.3797 | | | low |
| TCGA-G7-A8LE | TCGA | -1.775 | | | low |
| TCGA-UZ-A9PL | TCGA | -2.06413 | | | low |
| TCGA-GL-7773 | TCGA | -0.93797 | | | low |
| TCGA-G7-6792 | TCGA | -2.64419 | | | low |
| TCGA-Y8-A897 | TCGA | -2.98081 | | | low |
| TCGA-2Z-A9JS | TCGA | -3.19309 | | | low |
| TCGA-A4-8311 | TCGA | -3.05525 | | | low |
| TCGA-GL-A9DE | TCGA | -1.92301 | | | low |
| TCGA-F9-A97G | TCGA | -0.81615 | | | low |
| TCGA-A4-8098 | TCGA | -2.16785 | | | low |
| TCGA-BQ-5880 | TCGA | -0.22427 | | | low |
| TCGA-B3-3925 | TCGA | -2.16742 | | | low |
| TCGA-P4-AAVK | TCGA | -1.09325 | | | low |
| TCGA-KV-A6GE | TCGA | -1.8196 | | | low |
| TCGA-UZ-A9PU | TCGA | -2.71319 | | | low |
| TCGA-UZ-A9PJ | TCGA | -2.13857 | | | low |
| TCGA-GL-A9DC | TCGA | -2.7593 | | | low |
| TCGA-AL-3471 | TCGA | -1.88388 | | | low |
| TCGA-G7-6796 | TCGA | -0.56711 | | | low |
| TCGA-IZ-8196 | TCGA | -2.68896 | | | low |
| TCGA-B1-A655 | TCGA | -2.38423 | | | low |
| TCGA-UZ-A9PP | TCGA | -1.15861 | | | low |
| TCGA-GL-A9DD | TCGA | -2.43358 | | | low |
| TCGA-G7-7502 | TCGA | -1.3953 | | | low |
| TCGA-B1-A47N | TCGA | -2.31102 | | | low |
| TCGA-B3-8121 | TCGA | -2.44474 | | | low |
| TCGA-A4-7915 | TCGA | 0.666703 | | | high |
| TCGA-B9-A5W9 | TCGA | -1.64863 | | | low |
| TCGA-A4-7286 | TCGA | -3.19309 | | | low |
| TCGA-WN-A9G9 | TCGA | -1.05153 | | | low |
| TCGA-A4-8630 | TCGA | -2.98081 | | | low |
| TCGA-PJ-A5Z9 | TCGA | -2.3797 | | | low |
| TCGA-HE-7129 | TCGA | -2.71319 | | | low |
| TCGA-B9-4617 | TCGA | -2.68896 | | | low |
| TCGA-5P-A9JU | TCGA | -0.64176 | | | low |
| TCGA-BQ-5875 | TCGA | -1.16774 | | | low |
| TCGA-A4-8310 | TCGA | -2.59006 | | | low |
| TCGA-GL-A59T | TCGA | -3.19309 | | | low |
| TCGA-BQ-5881 | TCGA | -1.94504 | | | low |
| TCGA-KV-A6GD | TCGA | -1.10186 | | | low |
| TCGA-BQ-5878 | TCGA | -2.47668 | | | low |
| TCGA-AT-A5NU | TCGA | -3.19309 | | | low |
| TCGA-B3-A6W5 | TCGA | -3.19309 | | | low |
| TCGA-B1-7332 | TCGA | -2.3797 | | | low |
| TCGA-P4-A5EA | TCGA | 0.987287 | | | high |
| TCGA-BQ-5887 | TCGA | -0.54807 | | | low |
| TCGA-IZ-8195 | TCGA | -3.19309 | | | low |
| TCGA-G7-6795 | TCGA | -1.62019 | | | low |
| TCGA-2Z-A9J9 | TCGA | -0.45791 | | | low |
| TCGA-F9-A7Q0 | TCGA | -2.45055 | | | low |
| TCGA-B1-A656 | TCGA | -1.61607 | | | low |
| TCGA-A4-8517 | TCGA | -3.13729 | | | low |
| TCGA-A4-7828 | TCGA | 0.567764 | | | high |
| TCGA-A4-A48D | TCGA | -2.64586 | | | low |
| TCGA-2Z-A9JM | TCGA | -1.8196 | | | low |
| TCGA-G7-6793 | TCGA | 1.651061 | | | high |
| TCGA-UZ-A9PZ | TCGA | -3.19309 | | | low |
| TCGA-B3-3926 | TCGA | -2.11987 | | | low |
| TCGA-SX-A71W | TCGA | -3.19309 | | | low |
| TCGA-UZ-A9Q0 | TCGA | -2.45222 | | | low |
| TCGA-DW-7839 | TCGA | -0.94193 | | | low |
| TCGA-A4-A772 | TCGA | -3.19309 | | | low |
| TCGA-G7-A8LD | TCGA | 0.429966 | | | high |
| TCGA-2Z-A9JT | TCGA | -2.33381 | | | low |
| TCGA-HE-A5NF | TCGA | -1.95792 | | | low |
| TCGA-SX-A7SN | TCGA | -2.41724 | | | low |
| TCGA-5P-A9KF | TCGA | -2.3797 | | | low |
| TCGA-G7-7501 | TCGA | -0.13912 | | | low |
| TCGA-SX-A7SU | TCGA | -1.63136 | | | low |
| TCGA-HE-A5NJ | TCGA | -0.0124 | | | low |
| TCGA-A4-A5Y0 | TCGA | -2.04797 | | | low |
| TCGA-SX-A7SP | TCGA | -2.64586 | | | low |
| TCGA-SX-A71U | TCGA | -3.18021 | | | low |
| TCGA-5P-A9K6 | TCGA | -1.95792 | | | low |
| TCGA-AL-3473 | TCGA | -1.27395 | | | low |
| TCGA-P4-AAVL | TCGA | -0.78226 | | | low |
| TCGA-J7-6720 | TCGA | -1.93137 | | | low |
| TCGA-A4-A4ZT | TCGA | -1.68752 | | | low |
| TCGA-BQ-5882 | TCGA | -0.14984 | | | low |
| TCGA-A4-7287 | TCGA | 1.171161 | | | high |
| TCGA-BQ-5894 | TCGA | 1.199569 | | | high |
| TCGA-A4-A5XZ | TCGA | -3.19309 | | | low |
| TCGA-GL-A59R | TCGA | -3.19309 | | | low |
| TCGA-B3-4103 | TCGA | -2.3797 | | | low |
| TCGA-BQ-7048 | TCGA | -0.91949 | | | low |
| TCGA-Y8-A898 | TCGA | -2.3797 | | | low |
| TCGA-UZ-A9PQ | TCGA | -1.42973 | | | low |
| TCGA-G7-6790 | TCGA | -3.19309 | | | low |
| TCGA-BQ-7045 | TCGA | -0.48964 | | | low |
| TCGA-Y8-A895 | TCGA | -2.43358 | | | low |
| TCGA-DZ-6132 | TCGA | -1.59834 | | | low |
| TCGA-SX-A7SL | TCGA | -1.06648 | | | low |
| TCGA-HE-A5NL | TCGA | -3.19309 | | | low |
| TCGA-5P-A9JW | TCGA | -3.1472 | | | low |
| TCGA-A4-7734 | TCGA | -2.14173 | | | low |
| TCGA-5P-A9K2 | TCGA | -2.15627 | | | low |
| TCGA-BQ-5886 | TCGA | -2.48658 | | | low |
| TCGA-UZ-A9PV | TCGA | -2.38985 | | | low |
| TCGA-2Z-A9JR | TCGA | -2.64586 | | | low |
| TCGA-B1-A657 | TCGA | -2.34669 | | | low |
| TCGA-5P-A9JY | TCGA | -2.92501 | | | low |
| TCGA-A4-8516 | TCGA | -2.71319 | | | low |
| TCGA-UZ-A9PK | TCGA | -3.19309 | | | low |
| TCGA-IA-A83V | TCGA | -1.94504 | | | low |
| TCGA-DW-7834 | TCGA | -1.98525 | | | low |
| TCGA-5P-A9KE | TCGA | -3.19309 | | | low |
| TCGA-DW-7963 | TCGA | -3.19309 | | | low |
| TCGA-G7-6789 | TCGA | 1.651061 | | | high |
| TCGA-UZ-A9PN | TCGA | 0.183646 | | | low |
| TCGA-IZ-A6M8 | TCGA | -2.16596 | | | low |
| TCGA-5P-A9JV | TCGA | -0.62888 | | | low |
| TCGA-DZ-6135 | TCGA | -1.8308 | | | low |
| TCGA-IA-A83W | TCGA | -1.79779 | | | low |
| TCGA-5P-A9K3 | TCGA | 0.801388 | | | high |
| TCGA-BQ-5877 | TCGA | -1.06274 | | | low |
| TCGA-5P-A9K8 | TCGA | -1.40359 | | | low |
| TCGA-5P-A9KC | TCGA | -1.37683 | | | low |
| TCGA-DZ-6133 | TCGA | -2.3239 | | | low |
| TCGA-2Z-A9JO | TCGA | -1.30758 | | | low |
| TCGA-BQ-5888 | TCGA | -0.51696 | | | low |
| TCGA-A4-8515 | TCGA | -3.13729 | | | low |
| TCGA-BQ-5883 | TCGA | -0.48512 | | | low |
| TCGA-A4-8518 | TCGA | -1.1984 | | | low |
| TCGA-A4-8312 | TCGA | -0.20379 | | | low |
| TCGA-4A-A93X | TCGA | -1.56644 | | | low |
| TCGA-BQ-5885 | TCGA | -2.35593 | | | low |
| TCGA-GL-7966 | TCGA | 0.902712 | | | high |
| TCGA-G7-6797 | TCGA | -2.65707 | | | low |
| TCGA-A4-7585 | TCGA | 0.663546 | | | high |
| TCGA-Y8-A896 | TCGA | -1.87603 | | | low |
| TCGA-BQ-7046 | TCGA | -2.64586 | | | low |
| TCGA-G7-A4TM | TCGA | -1.83247 | | | low |
| TCGA-IA-A40X | TCGA | -2.65739 | | | low |
| TCGA-A4-7583 | TCGA | -2.92501 | | | low |
| TCGA-BQ-7059 | TCGA | -3.16008 | | | low |
| TCGA-AL-3472 | TCGA | -1.43634 | | | low |
| TCGA-A4-A5Y1 | TCGA | -1.39338 | | | low |
| TCGA-DW-7840 | TCGA | -2.3239 | | | low |
| TCGA-2Z-A9JQ | TCGA | -2.48658 | | | low |
| TCGA-B9-A44B | TCGA | 0.653781 | | | high |
| TCGA-B9-5156 | TCGA | -2.64586 | | | low |
| TCGA-P4-A5ED | TCGA | -1.72336 | | | low |
| TCGA-BQ-5879 | TCGA | 0.990444 | | | high |
| TCGA-HE-A5NI | TCGA | -1.53652 | | | low |
| TCGA-IA-A40U | TCGA | -1.95944 | | | low |
| TCGA-UZ-A9PR | TCGA | -2.60123 | | | low |
| TCGA-AL-A5DJ | TCGA | 0.379599 | | | high |
| TCGA-BQ-5893 | TCGA | 1.103831 | | | high |
| TCGA-DW-5560 | TCGA | 0.093876 | | | low |
| TCGA-PJ-A5Z8 | TCGA | -2.33381 | | | low |
| TCGA-BQ-7051 | TCGA | -3.19309 | | | low |
| TCGA-B3-4104 | TCGA | 0.611531 | | | high |
| TCGA-B9-A5W8 | TCGA | -3.19309 | | | low |
| TCGA-2Z-A9JK | TCGA | -0.50396 | | | low |
| TCGA-MH-A857 | TCGA | -0.50376 | | | low |
| TCGA-Y8-A8RZ | TCGA | -2.27226 | | | low |
| TCGA-V9-A7HT | TCGA | -3.16008 | | | low |
| TCGA-BQ-7044 | TCGA | -1.99645 | | | low |
| TCGA-Y8-A894 | TCGA | -0.94193 | | | low |
| TCGA-EV-5901 | TCGA | -1.94504 | | | low |
| TCGA-P4-A5E6 | TCGA | -1.97237 | | | low |
| TCGA-2Z-A9JE | TCGA | -2.71319 | | | low |
| TCGA-WN-AB4C | TCGA | -2.37366 | | | low |
| TCGA-SX-A7SS | TCGA | -1.84364 | | | low |
| TCGA-BQ-7062 | TCGA | -2.3797 | | | low |
| TCGA-MH-A562 | TCGA | -3.19309 | | | low |
| TCGA-2K-A9WE | TCGA | -2.89714 | | | low |
| TCGA-2Z-A9J8 | TCGA | -2.47668 | | | low |
| TCGA-2Z-A9J3 | TCGA | -0.7021 | | | low |
| TCGA-AL-3468 | TCGA | -1.89842 | | | low |
| TCGA-DW-7838 | TCGA | -0.84289 | | | low |
| TCGA-SX-A71S | TCGA | -2.3797 | | | low |
| TCGA-2Z-A9J5 | TCGA | -2.64586 | | | low |
| TCGA-2Z-A9JG | TCGA | -2.16596 | | | low |
| TCGA-UZ-A9PO | TCGA | -2.41724 | | | low |
| TCGA-2Z-A9J2 | TCGA | -2.51313 | | | low |
| TCGA-B1-A47M | TCGA | -2.92501 | | | low |
| TCGA-2Z-A9JD | TCGA | -1.69767 | | | low |
| TCGA-P4-AAVM | TCGA | -2.71174 | | | low |
| TCGA-F9-A8NY | TCGA | 1.618044 | | | high |
| TCGA-GL-A4EM | TCGA | -1.06884 | | | low |
| TCGA-MH-A55Z | TCGA | -3.13729 | | | low |
| TCGA-B9-4117 | TCGA | -1.81062 | | | low |
| TCGA-5P-A9KH | TCGA | -2.0577 | | | low |
| TCGA-DZ-6134 | TCGA | -1.91202 | | | low |
| TCGA-A4-7997 | TCGA | -1.34402 | | | low |
| TCGA-B9-5155 | TCGA | -2.55112 | | | low |
| TCGA-5P-A9K4 | TCGA | -2.44565 | | | low |
| TCGA-A4-7732 | TCGA | -2.74476 | | | low |
| TCGA-DW-5561 | TCGA | -1.63172 | | | low |
| TCGA-BQ-7050 | TCGA | -3.19309 | | | low |
| TCGA-2Z-A9J6 | TCGA | -2.3797 | | | low |
| TCGA-BQ-5884 | TCGA | -2.28995 | | | low |
| TCGA-DW-7836 | TCGA | -1.61579 | | | low |
| TCGA-2Z-A9JN | TCGA | -0.41644 | | | low |
| TCGA-HE-7130 | TCGA | -1.18622 | | | low |
| TCGA-AL-3466 | TCGA | 0.213349 | | | high |
| TCGA-A4-A7UZ | TCGA | -0.99686 | | | low |
| TCGA-BQ-5892 | TCGA | -0.44816 | | | low |
| TCGA-F9-A4JJ | TCGA | 0.649024 | | | high |
| TCGA-G7-A8LB | TCGA | -1.39026 | | | low |
| TCGA-Q2-A5QZ | TCGA | -1.11193 | | | low |
| TCGA-DW-7841 | TCGA | -1.84364 | | | low |
| TCGA-P4-A5E8 | TCGA | 0.666659 | | | high |
| TCGA-B9-A8YH | TCGA | -2.3797 | | | low |
| TCGA-SX-A7SM | TCGA | 0.353009 | | | high |
| TCGA-A4-7584 | TCGA | -2.71319 | | | low |
| GSM60246 | GEO | -0.73373 | | | low |
| GSM60265 | GEO | 0.567808 | | | high |
| GSM60258 | GEO | -0.69623 | | | low |
| GSM60255 | GEO | -0.85644 | | | low |
| GSM60270 | GEO | 0.059532 | | | low |
| GSM60259 | GEO | -0.06402 | | | low |
| GSM60268 | GEO | -1.51173 | | | low |
| GSM60250 | GEO | 0.254479 | | | high |
| GSM60247 | GEO | 0.215533 | | | high |
| GSM60260 | GEO | -1.84364 | | | low |
| GSM60254 | GEO | -1.46302 | | | low |
| GSM60261 | GEO | -2.6858 | | | low |
| GSM60263 | GEO | 0.241757 | | | high |
| GSM60257 | GEO | -2.3239 | | | low |
| GSM60262 | GEO | -0.27775 | | | low |
| GSM60251 | GEO | -2.3797 | | | low |
| GSM60264 | GEO | -0.50802 | | | low |
| GSM60252 | GEO | -1.98525 | | | low |
| GSM60267 | GEO | -2.6858 | | | low |
| GSM60253 | GEO | -0.84807 | | | low |
| GSM60271 | GEO | 1.171161 | | | high |
| GSM60249 | GEO | -1.06125 | | | low |
| GSM60273 | GEO | -0.79196 | | | low |
| GSM60245 | GEO | -1.7701 | | | low |
| GSM60272 | GEO | -0.37978 | | | low |
| GSM60274 | GEO | 0.278042 | | | high |
| GSM60248 | GEO | -2.60123 | | | low |
| GSM60256 | GEO | 1.171161 | | | high |

**Table S2 Detail information of the immune risk stratification**

| **pathway** | **p-value** | **adjusted**  **p-value** |
| --- | --- | --- |
| GO_MRNA_BINDING | 1.01E-06 | 0.001868 |
| GO_MICRO_RIBONUCLEOPROTEIN_COMPLEX | 1.01E-06 | 0.001868 |
| GO_MRNA_BINDING_INVOLVED_IN_POSTTRANSCRIPTIONAL_GENE_SILENCING | 1.06E-06 | 0.001868 |
| GO_OLFACTORY_RECEPTOR_ACTIVITY | 4.03E-06 | 0.005341 |
| GO_SENSORY_PERCEPTION_OF_SMELL | 6.04E-06 | 0.00633 |
| GO_KINETOCHORE | 1.06E-05 | 0.00633 |
| GO_DNA_DEPENDENT_DNA_REPLICATION | 1.23E-05 | 0.00633 |
| GO_APPENDAGE_MORPHOGENESIS | 1.24E-05 | 0.00633 |
| GO_MITOTIC_SISTER_CHROMATID_SEGREGATION | 1.25E-05 | 0.00633 |
| GO_EXTRACELLULAR_MATRIX_STRUCTURAL_CONSTITUENT | 1.42E-05 | 0.00633 |
| GO_APPENDAGE_DEVELOPMENT | 1.63E-05 | 0.00633 |
| GO_SISTER_CHROMATID_SEGREGATION | 1.76E-05 | 0.00633 |
| GO_CORNIFICATION | 1.78E-05 | 0.00633 |
| GO_CHROMOSOME_CENTROMERIC_REGION | 1.81E-05 | 0.00633 |
| GO_CELL_CYCLE_CHECKPOINT | 2.23E-05 | 0.00633 |
| GO_MESENCHYMAL_CELL_DIFFERENTIATION | 2.25E-05 | 0.00633 |
| GO_EAR_DEVELOPMENT | 2.27E-05 | 0.00633 |
| GO_PEPTIDASE_REGULATOR_ACTIVITY | 2.29E-05 | 0.00633 |
| GO_CONDENSED_CHROMOSOME | 2.34E-05 | 0.00633 |
| GO_DEVELOPMENT_OF_PRIMARY_SEXUAL_CHARACTERISTICS | 2.40E-05 | 0.00633 |
| GO_MEIOTIC_CELL_CYCLE | 2.92E-05 | 0.00633 |
| GO_STEM_CELL_DIFFERENTIATION | 2.94E-05 | 0.00633 |
| GO_HEART_MORPHOGENESIS | 2.96E-05 | 0.00633 |
| GO_NEGATIVE_REGULATION_OF_PEPTIDASE_ACTIVITY | 3.01E-05 | 0.00633 |
| GO_CELL_FATE_COMMITMENT | 3.17E-05 | 0.00633 |
| GO_NUCLEAR_CHROMOSOME_SEGREGATION | 3.26E-05 | 0.00633 |
| GO_SEX_DIFFERENTIATION | 3.53E-05 | 0.00633 |
| GO_DNA_REPLICATION | 3.60E-05 | 0.00633 |
| GO_CONDENSED_CHROMOSOME_CENTROMERIC_REGION | 3.64E-05 | 0.00633 |
| GO_ESTABLISHMENT_OF_PROTEIN_LOCALIZATION_TO_ENDOPLASMIC_RETICULUM | 3.68E-05 | 0.00633 |
| GO_MESENCHYME_DEVELOPMENT | 3.71E-05 | 0.00633 |
| GO_MITOTIC_NUCLEAR_DIVISION | 3.88E-05 | 0.006425 |
| GO_REGULATION_OF_CHROMOSOME_SEGREGATION | 4.04E-05 | 0.00648 |
| GO_REGULATION_OF_NUCLEAR_DIVISION | 4.20E-05 | 0.006548 |
| GO_CHROMATIN_ASSEMBLY | 4.40E-05 | 0.006649 |
| GO_ENDOPEPTIDASE_REGULATOR_ACTIVITY | 4.99E-05 | 0.007333 |
| GO_CHROMOSOME_SEGREGATION | 5.13E-05 | 0.007345 |
| GO_CHROMOSOMAL_REGION | 5.47E-05 | 0.007618 |
| GO_LIGAND_GATED_ION_CHANNEL_ACTIVITY | 5.70E-05 | 0.00774 |
| GO_NEGATIVE_REGULATION_OF_PROTEOLYSIS | 6.34E-05 | 0.008012 |
| GO_LYMPHOCYTE_MEDIATED_IMMUNITY | 6.40E-05 | 0.008012 |
| GO_GATED_CHANNEL_ACTIVITY | 6.46E-05 | 0.008012 |
| GO_REGIONALIZATION | 6.51E-05 | 0.008012 |
| GO_ADAPTIVE_IMMUNE_RESPONSE_BASED_ON_SOMATIC_RECOMBINATION_OF_IMMUNE_RECEPTORS_BUILT_FROM_IMMUNOGLOBULIN_SUPERFAMILY_DOMAINS | 6.68E-05 | 0.00804 |
| GO_MICROTUBULE_CYTOSKELETON_ORGANIZATION_INVOLVED_IN_MITOSIS | 6.94E-05 | 0.008171 |
| GO_TRANSCRIPTION_FACTOR_COMPLEX | 7.20E-05 | 0.008228 |
| GO_PEPTIDYL_TYROSINE_MODIFICATION | 7.30E-05 | 0.008228 |
| GO_COTRANSLATIONAL_PROTEIN_TARGETING_TO_MEMBRANE | 8.16E-05 | 0.008997 |
| GO_ROOF_OF_MOUTH_DEVELOPMENT | 8.53E-05 | 0.009045 |
| GO_FOREBRAIN_DEVELOPMENT | 8.54E-05 | 0.009045 |
| GO_MEIOTIC_CELL_CYCLE_PROCESS | 8.73E-05 | 0.009067 |
| GO_HORMONE_METABOLIC_PROCESS | 9.37E-05 | 0.009424 |
| GO_ENDOCRINE_SYSTEM_DEVELOPMENT | 9.43E-05 | 0.009424 |
| GO_GROWTH_FACTOR_RECEPTOR_BINDING | 9.78E-05 | 0.009568 |
| GO_MUSCLE_TISSUE_DEVELOPMENT | 0.000101 | 0.009568 |
| GO_RECEPTOR_COMPLEX | 0.000101 | 0.009568 |
| GO_NUCLEAR_TRANSCRIBED_MRNA_CATABOLIC_PROCESS_NONSENSE_MEDIATED_DECAY | 0.000105 | 0.009647 |
| GO_COLLAGEN_CONTAINING_EXTRACELLULAR_MATRIX | 0.000106 | 0.009647 |
| GO_DNA_BINDING_TRANSCRIPTION_ACTIVATOR_ACTIVITY | 0.000114 | 0.01018 |
| GO_MITOTIC_CELL_CYCLE_CHECKPOINT | 0.000115 | 0.01018 |
| GO_EXTRACELLULAR_STRUCTURE_ORGANIZATION | 0.000118 | 0.01018 |
| GO_DOUBLE_STRAND_BREAK_REPAIR | 0.000119 | 0.01018 |
| GO_EMBRYONIC_ORGAN_DEVELOPMENT | 0.000123 | 0.010243 |
| GO_REGULATION_OF_PEPTIDASE_ACTIVITY | 0.000124 | 0.010243 |
| GO_MUSCLE_CELL_DEVELOPMENT | 0.00013 | 0.010442 |
| GO_REPRODUCTIVE_SYSTEM_DEVELOPMENT | 0.000131 | 0.010442 |
| GO_CARDIAC_CHAMBER_DEVELOPMENT | 0.000132 | 0.010442 |
| GO_PATTERN_SPECIFICATION_PROCESS | 0.000137 | 0.010448 |
| GO_SKELETAL_MUSCLE_ORGAN_DEVELOPMENT | 0.000138 | 0.010448 |
| GO_DNA_RECOMBINATION | 0.000138 | 0.010448 |
| GO_INTEGRIN_BINDING | 0.000146 | 0.010883 |
| GO_MALE_SEX_DIFFERENTIATION | 0.000152 | 0.011153 |
| GO_ORGANELLE_FISSION | 0.000163 | 0.011831 |
| GO_B_CELL_MEDIATED_IMMUNITY | 0.000171 | 0.01226 |
| GO_CELL_SUBSTRATE_ADHESION | 0.000183 | 0.012926 |
| GO_EPITHELIAL_TO_MESENCHYMAL_TRANSITION | 0.00019 | 0.013265 |
| GO_LEUKOCYTE_MIGRATION | 0.000195 | 0.013386 |
| GO_SKELETAL_SYSTEM_MORPHOGENESIS | 0.000218 | 0.014805 |
| GO_REGULATION_OF_PROTEIN_SERINE_THREONINE_KINASE_ACTIVITY | 0.000226 | 0.015081 |
| GO_ENZYME_INHIBITOR_ACTIVITY | 0.000231 | 0.015081 |
| GO_DNA_BINDING_TRANSCRIPTION_REPRESSOR_ACTIVITY_RNA_POLYMERASE_II_SPECIFIC | 0.000231 | 0.015081 |
| GO_NEGATIVE_REGULATION_OF_BLOOD_VESSEL_ENDOTHELIAL_CELL_MIGRATION | 0.000255 | 0.016437 |
| GO_MESODERM_DEVELOPMENT | 0.00027 | 0.017118 |
| GO_STRUCTURAL_CONSTITUENT_OF_CYTOSKELETON | 0.000272 | 0.017118 |
| GO_POSITIVE_REGULATION_OF_CELL_ADHESION | 0.0003 | 0.018668 |
| GO_PASSIVE_TRANSMEMBRANE_TRANSPORTER_ACTIVITY | 0.000318 | 0.019565 |
| GO_EXTERNAL_SIDE_OF_PLASMA_MEMBRANE | 0.000333 | 0.020254 |
| GO_CELL_ADHESION_MOLECULE_BINDING | 0.000347 | 0.020896 |
| GO_DNA_INTEGRITY_CHECKPOINT | 0.000362 | 0.021521 |
| GO_GABA_RECEPTOR_COMPLEX | 0.000395 | 0.023226 |
| GO_CYTOSOLIC_RIBOSOME | 0.00041 | 0.023461 |
| GO_GLAND_DEVELOPMENT | 0.000412 | 0.023461 |
| GO_VIRAL_GENE_EXPRESSION | 0.000416 | 0.023461 |
| GO_MUSCLE_ORGAN_DEVELOPMENT | 0.000416 | 0.023461 |
| GO_OSSIFICATION | 0.000422 | 0.023514 |
| GO_SIGNAL_TRANSDUCTION_IN_RESPONSE_TO_DNA_DAMAGE | 0.00043 | 0.023701 |
| GO_EMBRYONIC_ORGAN_MORPHOGENESIS | 0.000445 | 0.024317 |
| GO_PRODUCTION_OF_MOLECULAR_MEDIATOR_OF_IMMUNE_RESPONSE | 0.000454 | 0.024517 |
| GO_EXTRACELLULAR_LIGAND_GATED_ION_CHANNEL_ACTIVITY | 0.000475 | 0.025401 |
| GO_CARDIAC_SEPTUM_DEVELOPMENT | 0.000492 | 0.026063 |
| GO_GROWTH_FACTOR_BINDING | 0.000513 | 0.026837 |
| GO_MITOTIC_SPINDLE_ORGANIZATION | 0.000517 | 0.026837 |
| GO_REGULATION_OF_CELL_SUBSTRATE_ADHESION | 0.000547 | 0.027632 |
| GO_MIRNA_MEDIATED_INHIBITION_OF_TRANSLATION | 0.000548 | 0.027632 |
| GO_REGULATION_OF_LYMPHOCYTE_ACTIVATION | 0.000548 | 0.027632 |
| GO_CHROMATIN_ASSEMBLY_OR_DISASSEMBLY | 0.000581 | 0.028924 |
| GO_COLLAGEN_TRIMER | 0.000584 | 0.028924 |
| GO_UROGENITAL_SYSTEM_DEVELOPMENT | 0.000594 | 0.029117 |
| GO_NEGATIVE_REGULATION_OF_HYDROLASE_ACTIVITY | 0.000604 | 0.029326 |
| GO_REGULATION_OF_IMMUNE_EFFECTOR_PROCESS | 0.000619 | 0.029545 |
| GO_EXTRACELLULAR_MATRIX_STRUCTURAL_CONSTITUENT_CONFERRING_TENSILE_STRENGTH | 0.00062 | 0.029545 |
| GO_INHIBITORY_EXTRACELLULAR_LIGAND_GATED_ION_CHANNEL_ACTIVITY | 0.000625 | 0.029545 |
| GO_REGULATION_OF_SISTER_CHROMATID_SEGREGATION | 0.000643 | 0.029927 |
| GO_HUMORAL_IMMUNE_RESPONSE_MEDIATED_BY_CIRCULATING_IMMUNOGLOBULIN | 0.000644 | 0.029927 |
| GO_RIBOSOME_BIOGENESIS | 0.000683 | 0.031458 |
| GO_CHROMOSOME_SEPARATION | 0.000704 | 0.032122 |
| GO_SNRNA_PROCESSING | 0.000762 | 0.034482 |
| GO_CONNECTIVE_TISSUE_DEVELOPMENT | 0.000822 | 0.036887 |
| GO_STARTLE_RESPONSE | 0.000838 | 0.037125 |
| GO_REGULATION_OF_CHROMOSOME_SEPARATION | 0.000846 | 0.037125 |
| GO_HEART_VALVE_DEVELOPMENT | 0.000848 | 0.037125 |
| GO_DNA_DAMAGE_RESPONSE_SIGNAL_TRANSDUCTION_BY_P53_CLASS_MEDIATOR | 0.000889 | 0.038508 |
| GO_FORELIMB_MORPHOGENESIS | 0.000908 | 0.038508 |
| GO_REGULATION_OF_ANIMAL_ORGAN_MORPHOGENESIS | 0.000918 | 0.038508 |
| GO_CARDIAC_VENTRICLE_DEVELOPMENT | 0.000921 | 0.038508 |
| GO_CARTILAGE_DEVELOPMENT | 0.000922 | 0.038508 |
| GO_COMPLEX_OF_COLLAGEN_TRIMERS | 0.000924 | 0.038508 |
| GO_DIGESTIVE_SYSTEM_DEVELOPMENT | 0.000935 | 0.038658 |
| GO_DORSAL_VENTRAL_PATTERN_FORMATION | 0.000956 | 0.039247 |
| GO_DIENCEPHALON_DEVELOPMENT | 0.000964 | 0.039247 |
| GO_NEGATIVE_REGULATION_OF_VASCULAR_ENDOTHELIAL_CELL_PROLIFERATION | 0.001039 | 0.041989 |
| GO_IMMUNOGLOBULIN_PRODUCTION | 0.001057 | 0.042406 |
| GO_CARDIAC_CHAMBER_MORPHOGENESIS | 0.001103 | 0.04391 |
| GO_CONDENSED_NUCLEAR_CHROMOSOME | 0.001152 | 0.045527 |
| GO_CHROMOSOME_LOCALIZATION | 0.001184 | 0.046447 |
| GO_REGULATION_OF_PEPTIDYL_TYROSINE_PHOSPHORYLATION | 0.001213 | 0.046725 |
| GO_ANTIGEN_BINDING | 0.001216 | 0.046725 |
| GO_NEURON_FATE_SPECIFICATION | 0.001218 | 0.046725 |
| GO_TRANSLATIONAL_INITIATION | 0.001264 | 0.048151 |

**Table S3 The top 10 upregulated and downregulated differentially expressed genes between high- and low- risk groups identified by the signature.**

| **Gene** | **Full gene name** | **Log_2_FC** | **FDR** |
| --- | --- | --- | --- |
| **Upregulated genes** | | | |
| RRM2 | ribonucleotide reductase regulatory subunit M2 | 7.344415578 | 1.02E-33 |
| BIRC5 | baculoviral IAP repeat containing 5 | 5.447034462 | 1.25E-31 |
| MELK | maternal embryonic leucine zipper kinase | 2.912125484 | 4.39E-31 |
| PRR11 | proline rich 11 | 2.509563335 | 5.70E-30 |
| DTL | denticleless E3 ubiquitin protein ligase homolog | 2.645117544 | 1.86E-26 |
| CEP55 | centrosomal protein 55 | 4.004752235 | 8.45E-26 |
| XK | X-linked Kx blood group | 1.573394222 | 1.14E-25 |
| OIP5 | Opa interacting protein 5 | 1.345198908 | 1.86E-25 |
| TOP2A | DNA topoisomerase II alpha | 12.50870005 | 1.86E-25 |
| PRC1 | protein regulator of cytokinesis 1 | 3.194241084 | 2.72E-24 |
| **Downregulated genes** | | | |
| PLA2G16 | phospholipase A and acyltransferase 3 | -49.05405998 | 9.38E-13 |
| C16orf86 | chromosome 16 open reading frame 86 | -3.856827789 | 1.78E-10 |
| UFSP2 | UFM1 specific peptidase 2 | -5.167889001 | 5.46E-10 |
| MTFR1L | mitochondrial fission regulator 1 like | -7.170903083 | 8.88E-10 |
| ZNF844 | zinc finger protein 844 | -2.819787664 | 3.07E-09 |
| OSCP1 | organic solute carrier partner 1 | -3.444590434 | 3.25E-09 |
| NICN1 | nicolin 1 | -5.519541366 | 3.70E-09 |
| SHMT1 | serine hydroxymethyltransferase 1 | -27.73351668 | 4.20E-09 |
| TCTA | T cell leukemia translocation altered | -16.41858653 | 7.92E-09 |
| SPRYD3 | SPRY domain containing 3 | -13.09143149 | 1.13E-08 |

Abbreviations: FDR, false discovery rate; FC, fold change
