# Supplementary figures and images for: Development and Validation of an Individualized Immune-Related Gene Pairs Prognostic Signature in Papillary Renal Cell Carcinoma
Source: Front Genet. 2020 Nov 9;11:569884. doi: 10.3389/fgene.2020.569884 (PMC7680997; doi:10.3389/fgene.2020.569884)

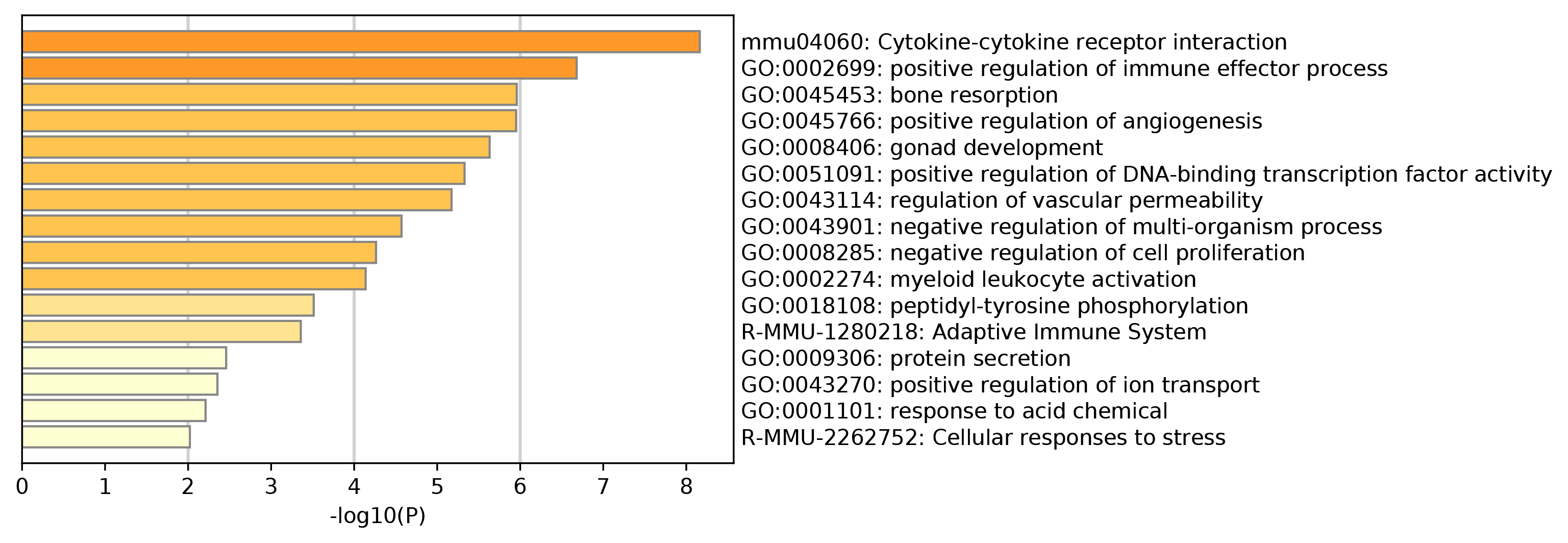

Supplement: Supplementary Figure 1 — Signaling pathways significantly enriched withing the 22 unique identified genes contained in the immune-related genes pairs. [file Image_1.TIF]

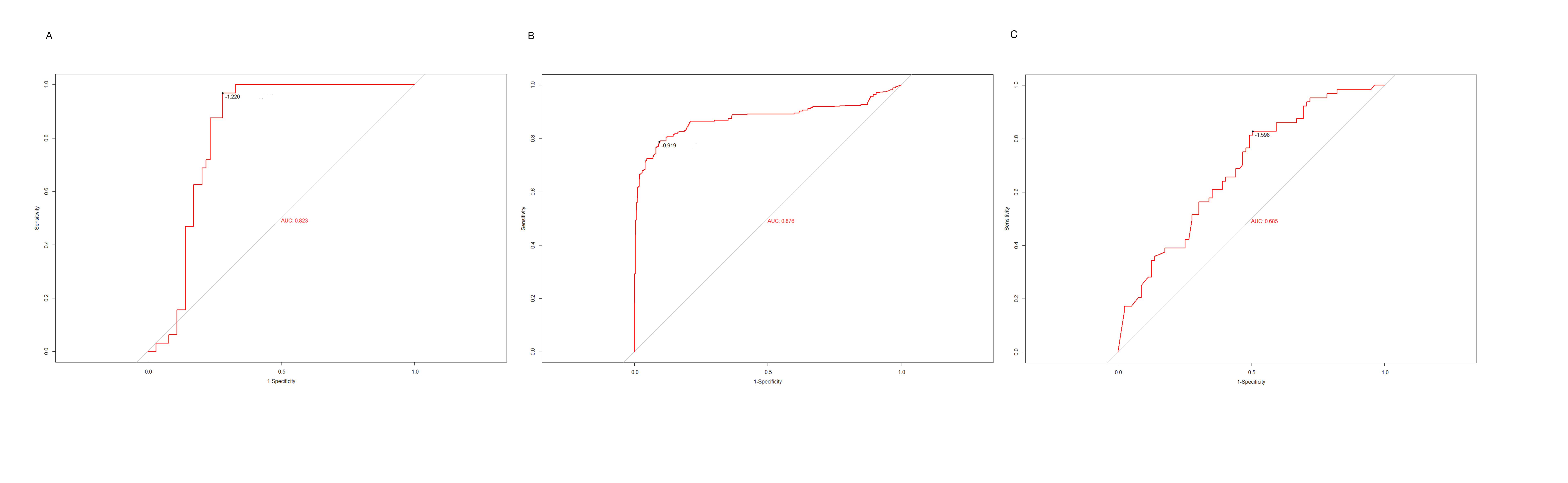

Supplement: Supplementary Figure 2 — Time-dependent receiver operating characteristic (ROC) curve for testing the diagnostic performance of the IRGPs signature on healthy samples vs. PRCC (a), ccRCC vs. PRCC (b), and type 1 PRCC vs. type 2 PRCC. PRCC, papillary renal cell carcinoma; ccRCC, clear cell renal cell carcinoma. [file Image_2.TIF]

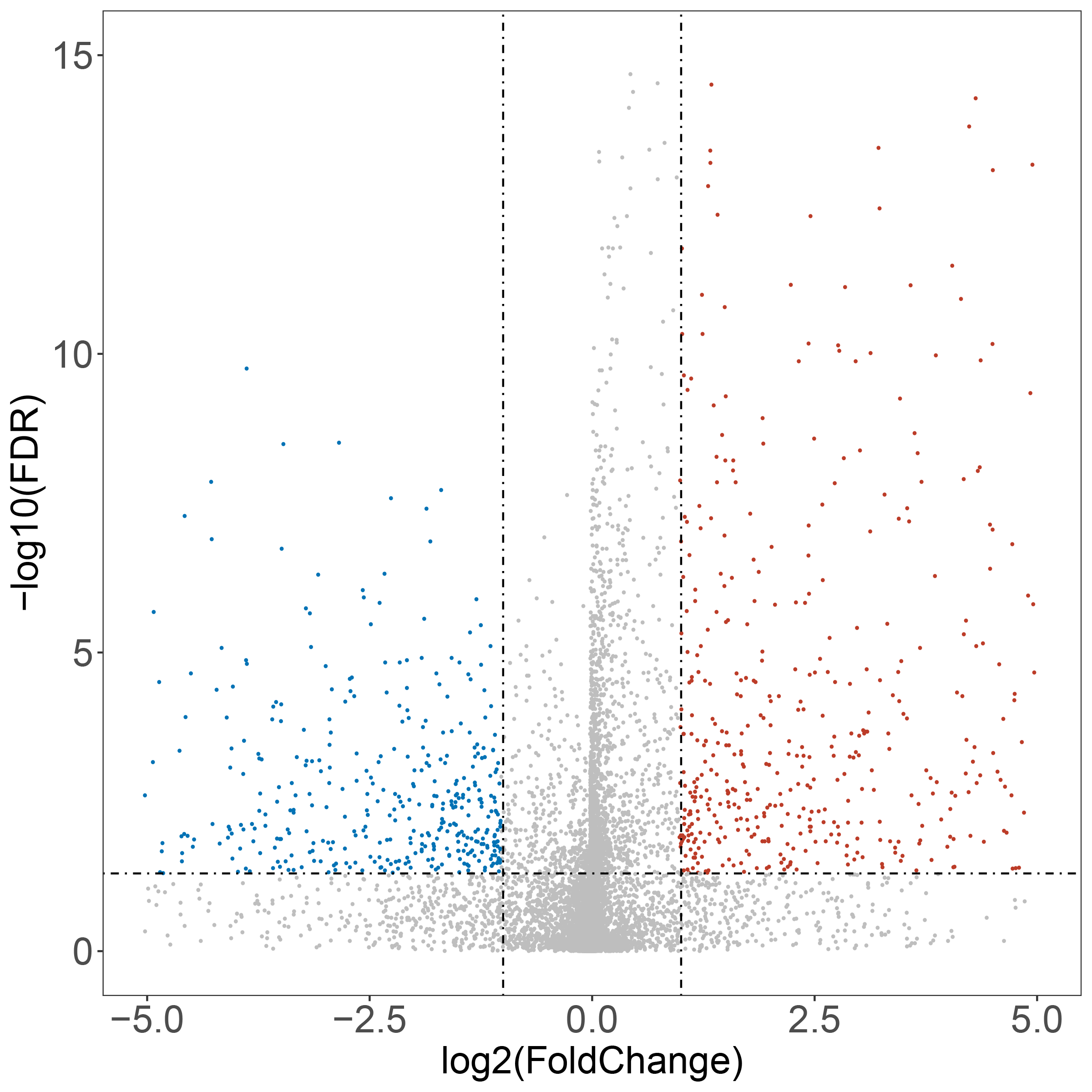

Supplement: Supplementary Figure 3 — Gene expression differences in high- and low-risk groups based on IRGP signature. The x-axis represents the difference between the two data sets following log2 conversion and the y-axis represents the p-value (log 10). [file Image_3.TIF]
